# Myeloid Cell-Derived Arginase in Cancer Immune Response

**DOI:** 10.3389/fimmu.2020.00938

**Published:** 2020-05-15

**Authors:** Tomasz M. Grzywa, Anna Sosnowska, Paweł Matryba, Zuzanna Rydzynska, Marcin Jasinski, Dominika Nowis, Jakub Golab

**Affiliations:** ^1^Department of Immunology, Medical University of Warsaw, Warsaw, Poland; ^2^Postgraduate School of Molecular Medicine, Medical University of Warsaw, Warsaw, Poland; ^3^Laboratory of Neurobiology BRAINCITY, Nencki Institute of Experimental Biology of Polish Academy of Sciences, Warsaw, Poland; ^4^The Doctoral School of the Medical University of Warsaw, Medical University of Warsaw, Warsaw, Poland; ^5^Laboratory of Experimental Medicine, Center of New Technologies, University of Warsaw, Warsaw, Poland; ^6^Genomic Medicine, Medical University of Warsaw, Warsaw, Poland; ^7^Centre of Preclinical Research, Medical University of Warsaw, Warsaw, Poland

**Keywords:** arginase, arginine, immunosuppression, tumor immunology, immunotherapy, T lymphocyte, T-cell metabolism

## Abstract

Amino acid metabolism is a critical regulator of the immune response, and its modulating becomes a promising approach in various forms of immunotherapy. Insufficient concentrations of essential amino acids restrict T-cells activation and proliferation. However, only arginases, that degrade L-arginine, as well as enzymes that hydrolyze L-tryptophan are substantially increased in cancer. Two arginase isoforms, ARG1 and ARG2, have been found to be present in tumors and their increased activity usually correlates with more advanced disease and worse clinical prognosis. Nearly all types of myeloid cells were reported to produce arginases and the increased numbers of various populations of myeloid-derived suppressor cells and macrophages correlate with inferior clinical outcomes of cancer patients. Here, we describe the role of arginases produced by myeloid cells in regulating various populations of immune cells, discuss molecular mechanisms of immunoregulatory processes involving L-arginine metabolism and outline therapeutic approaches to mitigate the negative effects of arginases on antitumor immune response. Development of potent arginase inhibitors, with improved pharmacokinetic properties, may lead to the elaboration of novel therapeutic strategies based on targeting immunoregulatory pathways controlled by L-arginine degradation.

## Introduction

The idea that the immune system can be harnessed to destroy tumors has been pursued for over a century (1). However, for decades the efforts have mainly focused on stimulating the immune system with recombinant cytokines, immune adjuvants, or co-stimulatory agonists that seemed critical for the induction of potent and sustained immune responses (1, 2). The rationale was that the immune system in cancer patients lacks sufficient power to mount anti-tumor response. It now seems however, that the interference with pathways dampening lymphocyte reactivity appears to be more effective in cancer patients than over-stimulation of effector mechanisms of immune system. The most successful approaches to impair tumor-elicited immunosuppressive mechanisms turned out to be monoclonal antibodies (referred to as immune checkpoint inhibitors) interfering with co-inhibitory molecules or their ligands, such as CTLA-4 (cytotoxic T-lymphocyte-associated protein 4), PD-1 (programmed cell death protein 1), or PD-L1 (programmed death-ligand 1). The spectacular therapeutic effects with unexpected ability to induce long-term tumor control led to clinical approval of checkpoint inhibitors (3–5).

Despite unprecedented antitumor efficacy, checkpoint inhibitors are effective in a minority of cancer patients, however. Thus, identification of response biomarkers as well as resistance mechanisms has become a priority for cancer researchers. A number of molecular mechanisms involved in the evasion of the anti-tumor immunity have been characterized in recent years (6). Central among them is the development of chronic inflammation (7, 8). Epidemiological data indicate that chronic inflammation is associated with poor prognosis (9). Mounting evidence indicates that the tumor microenvironment alters lymphoid and myeloid cells and converts them into potent immunosuppressive cells. It has become clear that tumor microenvironment, rich in inflammatory cells, is an indispensable component in the neoplastic process fostering proliferation, survival, and invasiveness of tumor cells (7). Chronic inflammation also triggers multiple regulatory pathways aimed at dampening immunity. The evolutionary rationale for this is to mitigate tissue damage and fibrosis. Coincidentally, the regulatory pathways impair development and/or activity of adaptive immune mechanisms that could be involved in eradication of tumor cells (8). Simultaneously, tumor cells frequently co-opt some of the signaling molecules participating in inflammation, such as adhesion molecules, cytokines, and growth factors for migration, invasion, and metastasis. Although there are many phenotypical and functional changes in different myeloid cell subpopulations, their precise role in the development of cancer resistance to immunotherapy is still not well-understood. This review will address the role of arginases (ARG), enzymes produced by tumor-infiltrating myeloid cells. The role of L-arginine (L-arg) metabolism in the regulation of immune response was of great interest in the 1980s and 1990s. However, further studies were focused mainly on L-arg-derived nitric oxide (NO) and its antimicrobial activity (10, 11), rather than immunosuppressive effects of L-arg deprivation. It is currently experiencing a renaissance due to increased awareness of the role of metabolic pathways in the regulation of immune cells function as well as due to the development of selective arginase inhibitors with improved pharmacokinetic properties. Novel tools and experimental models allowed to more precisely and comprehensively address the critical metabolic adaptations to microenvironmental changes experienced by immune cells. This is a clearly arginase-centered review, and it should be kept in mind that there are multiple other independent mechanisms of tumor immune evasion, including those affecting amino acids metabolism.

## Arginine and Arginases—Basic Biochemistry

**Figure 1:**
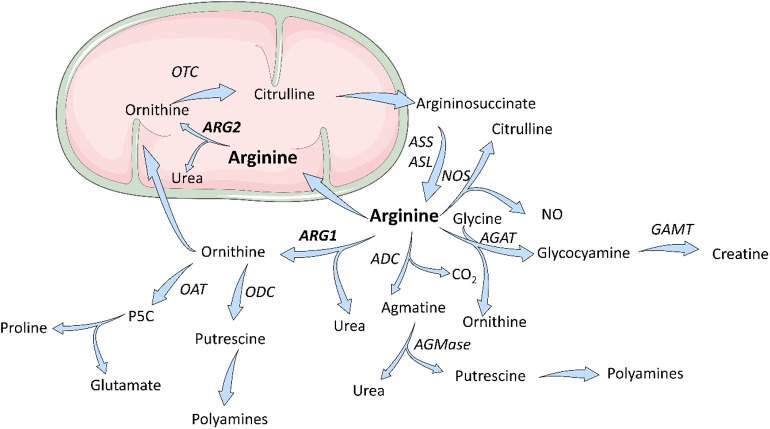
Scheme for arginine metabolism. In mammalian cells, L-Arginine is a substrate for four enzymes: ARG, NOS, ADC, AGAT. L-Arginine downstream metabolites are components of multiple metabolic pathways and are necessary for cells proliferation and collagen synthesis. ADC, arginine decarboxylase; AGAT, arginine:glycine amidinotransferase; AGMase, agmatinase; ARG, arginase; ASL, argininosuccinate lyase; ASS, argininosuccinate synthase; GAMT, guanidinoacetate N-methyltransferase; NOS, nitric oxide synthase; OAT; ornithine aminotransferase; OTC, ornithine transcarbamylase; P5C, pyrroline-5-carboxylic acid. Figure was modified from Servier Medical Art, licensed under a Creative Common Attribution 3.0 Generic License. http://smart.servier.com/.

L-arginine is a dibasic cationic amino acid participating in a variety of metabolic pathways (Figure 1) (12). There are three major sources of L-arg in the body—dietary intake, endogenous *de novo* production from L-citrulline or recycling, i.e., retrieval from degraded proteins. Under pathological conditions (bleeding, sepsis, trauma, cancer, or chronic inflammation) endogenous sources of L-arg become insufficient (13). Thus, L-arg is considered to be a semi-essential or conditionally-essential amino acid that in stressful conditions must be supplied in diet. Most of the endogenous L-arg synthesis is carried out in the kidney proximal tubules from intestinal L-citrulline (14). L-Arg plasma concentrations range between 50 and 250 μM (15–18) and are much lower than those in subcellular compartments (up to 1 mM) (19). In mammalian cells, L-arg transport through the plasma membrane is mediated by at least eight transporters (20). The uptake of L-arg occurs mainly via cationic amino acid transporters (CAT-1, CAT-2A, CAT-2B, and CAT-3, SLC7A1-3) (21). In human T-cells L-arg transport is mediated mainly by CAT-1 (22), while in myeloid cells by CAT-2 (23). Moreover, L-arg is transported through the plasma membrane by b^0, +^ AT (SLC7A9) and ATB^0, +^ (SLC6A14) that also transport neutral amino acids (20, 24, 25). L-type amino acid transporters γ^+^LAT1 (SLC7A7) and γ^+^LAT2 (SLC7A6) mediate mostly arginine export from the cells (20, 24). L-arg is metabolized in animal cells by four groups of enzymes, some of which exist in various isoforms. These include arginases, nitric oxide synthases (NOS), arginine decarboxylase (ADC), and arginine:glycine amidinotransferase (AGAT). Moreover, arginine deiminase (ADI) that hydrolyzes L-arg to L-citrulline and ammonia is expressed by some bacteria (26, 27). It is the first enzyme of the arginine dihydrolase system (ADS) that generates alkali and ATP for growth (28). These enzymes are encoded by arginine catabolic mobile element (ACME) (29) that was detected in *Staphylococcus aureus* and *Staphylococcus epidermidis* (30). L-arg metabolism by ADS enables survival in acidic environments, including human skin, disrupts host arginine metabolism, and contributes to the success of community-associated methicillin-resistant *S. aureus* (CA-MRSA) (31).

Arginases are manganese-containing enzymes that hydrolyze L-arg to L-ornithine and urea in the liver urea cycle (32). This is the most important pathway responsible for the conversion of highly toxic ammonia to excretable urea (33). L-Ornithine is a substrate for ornithine decarboxylase (ODC) that initiates polyamines synthesis, or it is metabolized by ornithine aminotransferase (OAT) to proline. Polyamines, such as putrescine, spermine, or spermidine are necessary for cell proliferation, while proline is necessary for collagen synthesis. Initially, it was thought that arginase is expressed only in the liver. However, further studies revealed that arginase is ubiquitously expressed in many types of cells (33), and that there are two different isoforms of this enzyme that catalyze the same biochemical reaction, but are expressed by different cells and are located in different cellular compartments. Human arginase 1 (ARG1) has 322 amino acids and is a cytosolic protein expressed primarily in the liver cells (34) as well as in the cells of the myeloid lineage (35). Human arginase 2 (ARG2) consists of 354 amino acids and can be found in mitochondria (36). It has ubiquitous expression, but usually at a lower level than ARG1. ARG2 has 58% sequence identity to ARG1 (37), but both enzymes are nearly identical within the catalytic region. There are also types of cells, such as endothelial cells, which have relatively high expression of both isoenzymes (38). The summary of the most important information on the two isoforms of arginase is presented in Table 1.

**Table 1:** Properties of the two arginase isoforms.

**Enzyme**	**Arginase 1**	**Arginase 2**
Genomic location in mouse	10; 10 A4	12; 12 C3
Number of amino acids	323	354
Genomic location in human	6q23	14q24.1
Number of amino acids	322	354
Sequence identity	ARG2 has 58% sequence identity to ARG1	
Structure	Homotrimer	
Catalyzed reaction	L-arg → urea + L-ornithine	
Localization	Cytosol	Mitochondrion
Tissue specificity	Liver, to a lesser extent kidney	Expressed ubiquitously, mainly kidney and prostate
Phenotype of knockout (KO) mice	Lethal, death occurs typically by postnatal day 17 (39). In conditional knockouts, death of adult mice occurs typically after 21 days of KO induction (40)	KO viable and apparently indistinguishable from wild-type mice (41, 42)
Phenotype of deficiency in humans	Urea cycle disorder, hyperargininemia, progressive neurologic impairment (43)	Defects not described.ARG2 level is increased in ARG1-deficient patients (43, 44)
Effect of ARG on immune response	Immunosuppression (45)	Unclear - immunosuppression (46–49), but also expressed by proinflammatory cells (50–52)

An important metabolic pathway of L-arg involves the activity of NOS. There are three isoforms of this enzyme—neuronal (nNOS or NOS1), inducible (iNOS or NOS2) and endothelial (eNOS or NOS3). NOS2 can be induced in many types of cells, but when present in activated myeloid cells it produces NO at a very high rate. There are multiple layers of competition between NOS2 and ARG1 in myeloid cells and both enzymes are induced by cytokines regulating different types of the immune response. NOS2 in myeloid cells is induced by type 1 cytokines (mainly IFN-γ), while ARG1 expression is regulated by IL-4 and IL-13. Considering that *K*_*m*_ of ARG1 is ~1,000-fold higher than that of NOS2, the intracellular L-arg could be expected to be mainly metabolized to NO, rather than to L-ornithine and urea. However, *V*_*max*_ of NOS is three orders of magnitude slower than that of ARG1 (53, 54). Thus, both enzymes compete for the same substrate. Intriguingly, insufficient L-arg concentrations lead to NOS uncoupling, whereby rather than NO these enzymes generate superoxide anions. Superoxide then rapidly reacts with any available NO molecules to form peroxinitrites that further decrease NOS activity by oxidizing tetrahydrobiopterin (BH_4_) (54). Moreover, induction of ARG1 that limits L-arg availability is involved in the regulation of NOS2 expression as L-arg is necessary for the translation of NOS2-encoding mRNA (55).

During acute wound healing resident myeloid cells express high levels of NADPH oxidase (NOX2) and NOS2, which participate in normal antimicrobial defense mechanisms by producing superoxide anion and NO, respectively. Then, after 3–5 days, a repair phase is initiated, which is associated with the appearance of ARG1^+^ macrophages. L-arg degradation produces L-ornithine that is converted by OAT to L-proline used as a substrate in collagen synthesis (56). ODC converts L-ornithine to polyamines that stimulate cell proliferation. This highly regulated process is perpetuated in tumors that are frequently described as wounds that never heal (57).

## Arginine and Arginase in Tumors

Tumor progression is associated with alterations in metabolic pathways in tumor cells as well as in the cells forming the tumor microenvironment. Altered metabolic phenotype of tumors includes changes in L-arg concentrations. For example, the concentration of L-arg in the core regions of solid tumors is about 5 times lower as compared with tumor periphery and this difference turned out to be the highest among all of the measured amino acids (58). Quantification of interstitial fluid metabolites in murine tumors has also revealed that L-arg is the most strongly depleted amino acid in the tumor microenvironment (59). The mechanisms of L-arg depletion are incompletely elucidated. On the one hand, L-arg can be consumed by tumor cells that have increased metabolic demands and use it for protein synthesis, but it can also be used by enzymes such as arginases or NOS. Many studies reported that arginases can be produced by tumor cells (46, 60, 61), but even larger number of reports indicate that the major L-arg-metabolizing cells are found in the tumor stroma. It has not been studied in sufficient detail as to which cells in the tumor environment are mainly responsible for L-arg depletion. It is also entirely possible that this process is highly variable and changes in the course of tumor progression, with tumor cells or stromal cells predominating in L-arg metabolism at various stages of neoplastic disease.

### Arginase and Tumor Prognosis

High ARG expression and activity have been reported in many types of human cancers, but its role as a prognosis factor remains vastly undetermined and usually studied on small populations of patients. Moreover, drawing conclusions from the limited number of studies is further complicated by a lack of standardized criteria for ARG measurements. For example, different cutoff criteria were applied to groups of patients with “low arginase” and “high arginase” expressing tumors, or studying either ARG1 or ARG2 expression profiles. Nonetheless, increasing evidence shows that overexpression of ARG1/2 (with or without subsequent decline in serum L-arg concentrations) should be perceived as a poor prognostic factor in a wide variety of cancer types including head and neck cancer (62), neuroblastoma (46), acute myeloid leukemia (AML) (61), pancreatic ductal carcinoma (63), ovarian carcinoma (64), or colorectal cancer (65). High expression of ARG1 in hepatocellular carcinoma also seems to play a role as a negative predictive factor that correlates with shorter median time to recurrence (66) and more aggressive tumors (67), but further evidence is required to support these observations as a contradictory report exists (68).

Although a number of studies provide strong evidence for increased ARG activity in both tissue (69) and blood (70–72) obtained from patients with breast cancer, so far no study was conducted to establish the role of ARG activity in determining the prognosis of breast cancer patients. Notably, contradictory reports exist that show a decrease in blood plasma ARG activity in breast cancer patients, however, these are based on very limited number of enrolled patients (73, 74). Similarly to breast cancer, increased ARG activity was found in skin (75), cervical (76), thyroid follicular (77), thyroid papillary and follicular variant of papillary (77), gastric, bile duct (78), and esophageal (79) cancers. However, again no study exists in these types of cancers that would demonstrate the impact of ARG activity/abundance on patients' prognosis.

Finally, there are tumors such as prostate (80–82) and lung cancer (83) as well as tumors that are auxotrophic for L-arg (these are not capable of re-synthesis of L-arg from citrulline due to the lack of expression of argininosuccinate synthetase-1, ASS-1), such as melanoma (84) and renal carcinoma (85, 86), where no correlation between ARG levels and survival has been found.

A critical question arises whether ARG in tumors is produced by tumor cells or by tumor-associated stromal cells that include mesenchymal as well as immune cells, among which myeloid cells seem to be the main source of the enzyme. Regrettably, no studies have been conducted that would directly address this issue and whether this is of any significance for cancer patients survival, whether ARG is expressed by tumor or tumor-infiltrating myeloid cells.

## Arginase in Tumor-Infiltrating Myeloid Cells

**Figure 2:**
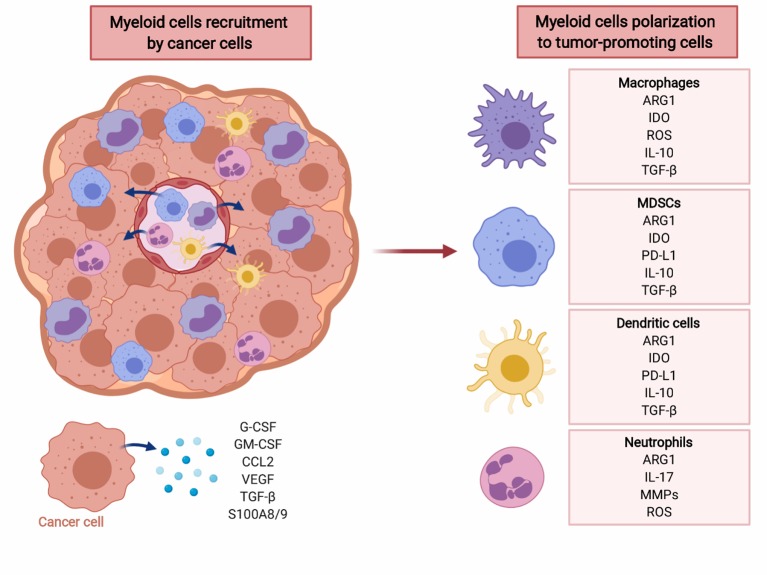
Cancer cells recruit myeloid cells to tumor microenvironment (TME) and induce their polarization to immunosuppressive phenotype. Myeloid cells, including macrophages, MDSC, dendritic cells, and neutrophils create tumor-promoting, immunosuppressive TME via multiple factors including reactive oxygen species (ROS), cytokines (IL-10, TGF-β), PD-L1, as well as ARG1. Created with BioRender.

Myeloid cells are major contributors to immune defense against pathogens and play an important role in tissue remodeling. During acute infections GM-CSF drives myelopoiesis in the bone marrow, and G-CSF as well as M-CSF induce further differentiation of granulocytes and macrophages, respectively (87). Some tissue macrophages develop from embryonic precursors that directly home to peripheral tissues and become a self-renewing population (88). Mature myeloid cells are specialized in killing infectious microorganisms and play an important role in promoting development of adaptive immunity. However, in cancer and other chronic inflammatory conditions constant production of low concentrations of myeloid growth factors and various inflammatory mediators dysregulate myeloid cells differentiation (Figure 2) (89–94). It is currently not well-understood what events trigger this disturbed myelopoiesis, but it must be emphasized that this process evolves over many years of tumor development and likely involves multiple independent mechanisms. Some of these might be completely stochastic, but in the course of tumor progression become promoted in a trial-and-error process that selects for mechanisms that best fit the demands of growing tumors.

Myeloid cells, especially tumor-infiltrating myeloid cells (TIMs), are a highly heterogeneous population (95). TIMs include monocytes, macrophages, dendritic cells, granulocytes, mast cells, as well as their immature precursors that have not completed their differentiation processes. The latter cells are normally found in the bone marrow, but in the course of tumor development they frequently expand and relocate to the spleen, lymph nodes and the tumor itself, and can be found at increased numbers in the peripheral blood (96, 97). These cells express immune checkpoint molecules, deplete essential metabolites, release immunosuppressive adenosine and its metabolites, produce reactive oxygen species, secrete immunoregulatory cytokines, growth-promoting, and proangiogenic factors (Figure 2). Moreover, they induce various populations of regulatory T-cells that impair antitumor immune response (98). Due to their strong immunosuppressive functions these cells have been termed myeloid-derived suppressor cells (MDSCs). There are two major subsets of MDSCs—monocytic (M-MDSC) and granulocytic (polymorphonuclear, PMN-MDSC) (99). Both have been associated with dysregulation of immune response in murine cancer models and in cancer patients, although still the majority of studies report the suppressive potential of total MDSCs (100). In mouse tumor models that mostly involve transplantation of tumor cells, the expansion of MDSCs is very rapid. This is in contrast to slow-growing tumors, including diethylnitrosoamine (DEN)-induced or MYC-expressing hepatocellular carcinoma, that in terms of the rate of tumor progression more accurately reflect human cancer (101). In many types of humans tumors, including lung, colon, uterus, cervix, bladder, or thyroid gland cancers, the increased numbers of M-MDSCs in peripheral blood correlate with worse clinical outcomes (102). In melanoma or liver cancer, however the increased numbers of both PMN-MDSCs and M-MDSCs were associated with poorer outcomes (102), while in renal cell carcinoma PMN-MDSCs seem to predominate (103). Importantly, increased numbers of MDSCs are observed also in patients with pancreatic premalignancy—intraductal papillary mucinous neoplasm (IPMN), and in patients with colon adenomas, as compared with healthy controls (97).

Nearly all myeloid cells have been shown to produce ARG1 in mice (Figure 2). However, there are substantial differences in the expression of arginases by myeloid cells between mice and men (104). In humans, arginase is produced mainly by granulocytes and no arginase activity is detectable in monocytes, macrophages nor dendritic cells (105). The differences in expression of both isoforms of arginase by myeloid cells in mice and humans is summarized in Table 2.

**Table 2:** Differences in arginases expression in myeloid cells between mouse and human.

**Type of cell**	**Arginase 1**				**Arginase 2**			
	**Mouse**		**Human**		**Mouse**		**Human**	
Monocytes	+	(106)	–^c^	(107, 108)	+	(109)	+	(50, 110)
Macrophages	+^a^	(111)	–^c^	(105)	+^b^^,^^c^^,^^d^	(36, 112)	+	(113)
M2 macrophages	+^a^	(114)	–^c^	(108)	**–**^d^^,^^e^	(51)	**–**^e^	(50)
TAMs	+	(115)	–^f^	(116)	+	(117, 118)	+	(63)
MDSCs	+^a^	(103)	+	(103)	+ –	(119, 120)	+	(121)
Neutrophils	+	(122)	+	(105, 123)	+	(117, 124)	+	(113)
Dendritic cells	+^a^	(112)	**–**^c^	(105)	+ –	(112, 125)	+	(126)

a *Induced by Th2 cytokines*.

b *Not significantly modulated by Th1 cytokines*.

c *Not significantly modulated by Th2 cytokines*.

d *Induced by LPS*.

e *ARG2 is proposed as a marker of proinflammatory M1 macrophages (50–52, 127)*.

f *ARG1 expression in human TAMs was minimal and on the same level as in control tissue-resident macrophages (116)*.

+, *expression*; –, *undetectable or very low expression; TAMs, tumor associated macrophages; MDSCs, myeloid-derived suppressor cells*.

The first report linking immunosuppression with arginase activity in macrophages was published over 40 years ago (128). However, the concept that L-arg metabolism is associated with regulation of the immune response did not gain much attention initially. It was suggested that suppressive effect of arginase may be just an interesting problem of *in vitro* culture (129). However, soon other studies described depletion of L-arg by macrophages expressing arginase both *in vitro* (130) and *in vivo* in tumor-bearing mice (131). The authors hypothesized that arginase may be an effector mechanism of macrophages against infectious microorganisms and tumor cells (131). After over 30 years we know that arginase plays an opposite role in immune response and is one of the main mechanism of immunosuppression.

**Figure 3:**
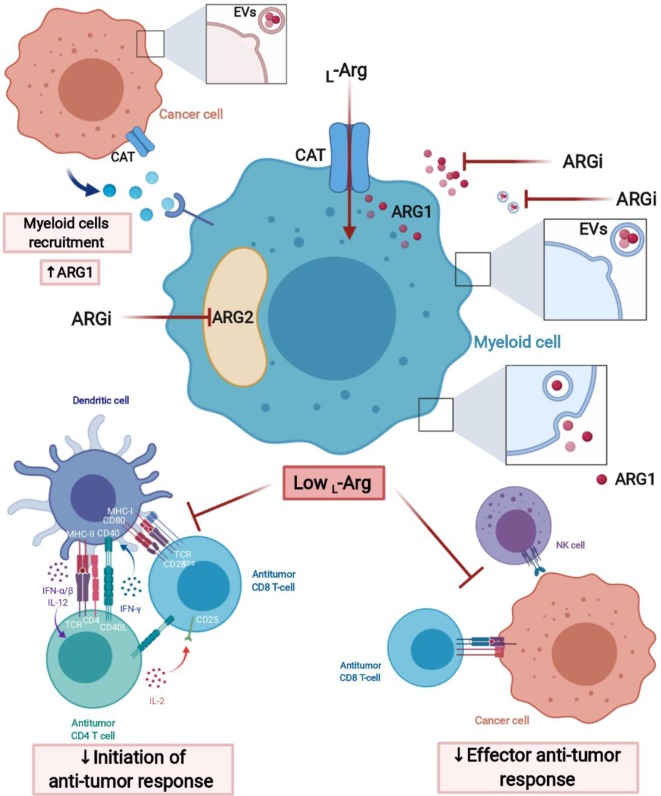
L-Arginine-depleting arginases lead to the impaired anti-tumor response. Arginases may act intracellularly (cytoplasmic ARG1 and mitochondrial ARG2) and extracellularly (secreted ARG1) leading to the local depletion of L-arginine in tumor microenvironment (TME). Moreover, ARG1 may have effects in sites distant from the TME, when packed into extracellular vesicles (EVs), transported over long distance and internalized by myeloid cells, for instance, in tumor-draining lymph node. Arginase inhibitors (ARGi) should target both isoforms (ARG1 and ARG2) and easily penetrate the cell membrane to block extracellular and intracellular arginases, as well as arginase in EVs. Created with BioRender.

L-arg depletion by suppressive myeloid cells in the tumor microenvironment can occur by increased L-arg uptake by CAT-2B transporters (132), which is followed by arginase-mediated hydrolysis (Figure 3). Myeloid cells also secrete arginase to the microenvironment (133), where it acts mainly locally due to short circulating half-life (134). Murine MDSCs deplete L-arg by increased uptake and intracellular degradation, in contrast to human MDSCs that mainly release arginase into the circulation (103). ARG1 may also be secreted in extracellular vesicles (EVs) by MDSCs (135). In EVs, arginase remains stable and may exert greater than local effects, for instance in draining lymph nodes (64).

### Arginase in Myeloid-Derived Suppressor Cells

MDSCs have been the most intensively studied cells in terms of L-arg metabolism. Bronte et al. were the first to show that myeloid cells accumulating in the spleens of tumor-bearing mice express ARG1 and suppress the proliferation of allogeneic T-cells (141). Liu et al. showed that myeloid cells in the tumor microenvironment express arginase and suppress cytotoxic T lymphocyte (CTL) activity in NO-independent manner (142). Since then, many other studies confirmed that immature tumor myeloid cells express ARG1 in mice and humans with cancer and that the activity of this enzyme is involved in suppression of T-cell response (132, 143–146). The majority of studies indicate that arginase plays a more important role in PMN-MDSC rather than M-MDSC (103, 147–149). However, the role of this enzyme in the regulatory activities of the latter cells should not be completely dismissed. For example, iNOS inhibitor together with ARG inhibitor diminished the suppression driven by M-MDSC, with no effect on PMN-MDSC (150).

In humans, PMN-MDSCs store ARG1 in granules and release it to the extracellular milieu (103). It leads to the depletion of L-arg and suppression of anti-tumor response. In patients with pancreatic ductal adenocarcinoma CD13^hi^ PMN-MDSCs were identified that produce ARG1 and suppress alloreactive T-cell responses in ARG1-dependent manner. Patients with more CD13^hi^ PMN-MDSCs had significantly shorter survival than those with predominant CD13^low^ PMN-MDSCs in the tumor infiltrates (149). Similarly, ARG1-producing MDSCs in patients with renal cell carcinoma turned out to be of granulocytic lineage (103). Interestingly, treatment of patients with IL-2 increased the number of these cells in peripheral blood, as well as in the plasma concentrations of ARG1 (103). Whole mount labeling and clearing followed by three-dimensional light sheet microscopy of head and neck carcinomas identified intratumoral hotspots of PMN-MDSCs that co-localized with T-cells. Those T-cells that were in close proximity to ARG1-positive PMN-MDSCs had strongly reduced expression of granzyme B (serpin participating in cytotoxic effects of T-cells) and Ki67 (a proliferation marker) (151). In multiple myeloma IL-18 was shown to induce ARG1^+^ PMN-MDSCs that suppress immune response (152). In KRAS^G12D^ genetically engineered mice that develop lung tumors resembling NSCLC, PMN-MDSCs were observed to cause T-cell suppression by L-arg depletion. Arginase inhibitor has not only restored T-cell function, but caused significant regressions of tumors in these mice (117). Arginase-expressing MDSCs were also shown to induce Tregs in murine tumor models (153) as well as in cancer patients (154). In some of these studies, this effect was abrogated by arginase inhibitor (153) indicating a specific role of this enzyme in Treg development (see below).

**Figure 4:**
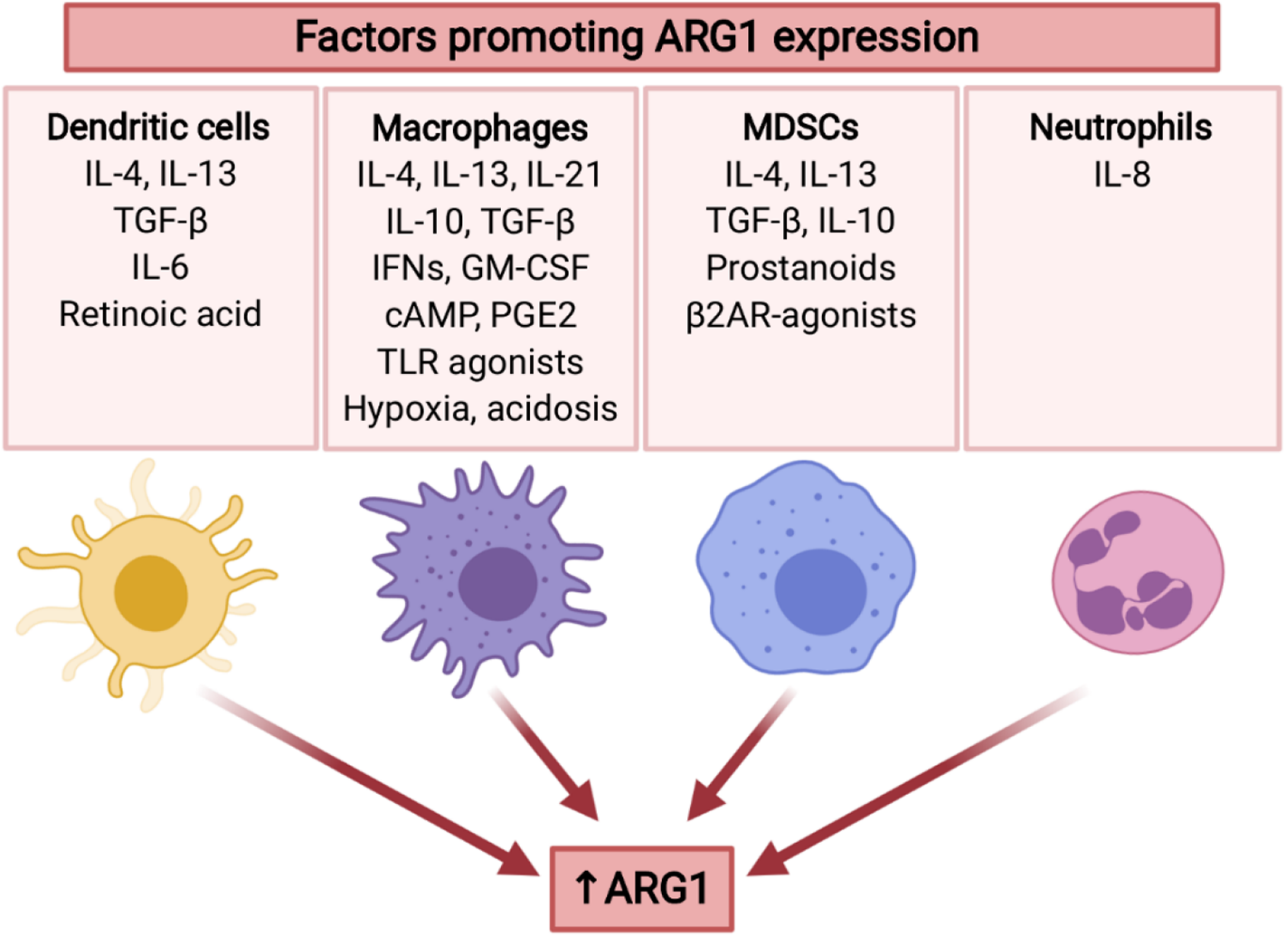
Several tumor-associated factors promote ARG1 expression in myeloid cells. ARG1 expression is mainly induced by type 2 cytokines (IL-4, IL-13), as well as immunosuppressive cytokines (TGF-β, IL-10). Moreover, it may be promoted by TME factors including hypoxia and acidosis, as well as stress mediators. ARG1 expression is also induced by GM-CSF (136), TLR agonists (137), and cAMP (138). IL-10 and IL-21 increase IL-4-induced ARG1 expression (139, 140). Created with BioRender.

Arginase expression in tumor MDSCs is increased as compared with the cells of the same phenotype isolated from spleen (155). Both inflammatory and tumor-derived factors are involved in the regulation of ARG1 expression in MDSCs (Figure 4). For example, tumor-infiltrating MDSCs stimulated with TGF-β and IL-10 demonstrated high ARG1 activity (156). One mechanism involves stress sensor C/EBP-homologous protein (CHOP), which directly activates ARG1 gene through inhibition of LIP transcription suppressor. CHOP expression in MDSCs is induced by ROS and further by the activating-transcription factor-4 (ATF-4) (157). Intriguingly, diminished L-arg concentrations have been shown to induce accumulation of arginase-expressing MDSCs in the tumors after administration of pegylated recombinant ARG1 to tumor-bearing mice (158) indicating potential threats associated with L-arg-depleting therapeutic strategies for cancer. ARG1 levels in MDSCs from patients with head and neck cancer were regulated by STAT3 signaling (159). Accordingly, STAT3 silencing in MDSCs from prostate cancer patients abrogated their immunosuppressive activity (160). Chronic stress, which frequently accompanies cancer, was reported to increase the generation of ARG1^+^ MDSCs in mice and humans, through catecholamines stimulating β2 adrenergic receptors (β2AR). Induction of ARG1 by isoproterenol (a β2AR agonist) was associated with STAT3 phosphorylation in MDSCs (161). It was found that prostanoids produced by COX2 are responsible for mediating ARG1 overexpression in MDSCs by lung cancer cells in *in vitro* and *in vivo* models (162). The mechanism of ARG1 upregulation in MDSC is probably controlled by EP4 receptor for PGE_2_. Those findings were confirmed in other tumors (144, 163). MDSC not only infiltrate tumor and its environment, they were also found in peripheral blood. (103). MDSC abundance in blood correlated with staging in HNSCC patients. Moreover, MDSC in HNSCC have high level of pSTAT3 and ARG1 and potently inhibit T-cells proliferation (159).

### Macrophages

Macrophages are the main phagocytic population of cells within tumors (47). However, contrary to their natural role in promoting immunity against infectious microorganisms, tumor-associated macrophages (TAMs) are involved in promoting tumor progression, partly through creating an immunosuppressive microenvironment (47). The majority of reports on macrophages in cancers describe their function in the context of *in vitro* polarization into M1 or M2 subsets (164). This classification is currently not recommended as TAMs are represented by a continuum of phenotypic variants (47), but will be incidentally used hereafter considering that the existing literature specifically refers to M1 and M2 macrophage subsets. It must be underscored however, that TAMs are highly diverse and form a wide range of populations with various functional roles (165). Additionally, these cells do not form a stable population, but are highly variable both in time and location within the tumor milieu (165, 166). So called M1 macrophages are induced by lipopolysaccharide (LPS) and type 1 cytokines (mainly IFN-γ), express high levels of tumor necrosis factor (TNF), IL-12, iNOS, and MHC class II molecules and are considered to participate in anti-tumor immunity (47). M2 macrophages are induced by type 2 cytokines and express ARG1, IL-4, IL-13, IL-10, and CD206 (47, 167). Cytokines, especially those associated with type 2 immune response (IL-4 and IL-13) that activate the transcription factors STAT6, PU.1, and CCAAT/enhancer binding protein β (C/EBPβ) were shown to directly induce signaling pathways leading to increased production of ARG1 in macrophages (168). IL-4- and IL-13-activated STAT6 with STAT3 and C/EBPβ bind to an enhancer in the ARG1 locus (169). Some cytokines, including IL-10 and IL-21, upregulate the expression of IL-4Rα and IL-13Rα1, leading to the increased IL-4-induced ARG1 expression (139, 140). M2 macrophages are the most abundant population of myeloid cells in tumors, and their presence is usually associated with poor prognosis, tumor cell invasion, metastasis, and neovascularization (170, 171). Importantly, TAMs are considered to be of either embryonic origin or to derive from hematopoietic stem cells (HSCs) (172–175). Both populations are found in the tumors in approximately similar ratio, but it seems that it is mainly the latter population that includes cells with immunosuppressive properties (47). HSC-derived macrophages in a tumor microenvironment sense local physicochemical conditions that are different than in many normal tissues and include hypoxia, acidosis, changes in the composition of extracellular matrix proteins (that affects rigidity of the tumor tissue), nutrient insufficiency, different cellular metabolites, various growth factors and inflammatory mediators (prostanoids, cytokines, etc.) (165). Necrosis and other forms of cell death lead to appearance of cell debris as well as cell death-associated molecular patterns [CDAMPs, also known as death-associated molecular patterns—DAMPs (176)] that additionally affect differentiation of macrophages. Many of these environmental conditions have been shown to induce ARG1 in TAMs including hypoxia via hypoxia-inducible factors (HIFs) (177), lactic acid (in a HIF-1α-dependent mechanism) (178), or COX2 via prostaglandin E_2_ (162) (Figure 4). Even local acidosis might be involved in ARG1 induction as resting macrophages at pH of 6.1 were observed to induce expression of VEGF, HIF-1α and ARG1 (179), and induction of ARG1 by IL-4 was stronger at pH of 6.8 (180). Cancer-associated fibroblasts (CAFs) have been shown to regulate macrophage differentiation and confer these immunosuppressive cells with the ability to secrete high levels of IL-6 and to produce collagen that leads to the development of tumor desmoplasia (181). Collagen forms a scaffold for many secreted mediators including TGF-β. The number of ARG1 positive macrophages was decreased in Mer tyrosine protein kinase (MERTK) knock-out mice (182). MERTK is involved in signaling triggered by recognition of apoptotic cells. Quite unexpectedly, a recent study revealed that type I interferons (IFNs) inhibit monocyte to macrophage differentiation within tumor and induce strong expression of ARG1 (183).

Macrophages are the main source of ARG1 within tumors in a murine model of colon adenocarcinoma (115). *In vivo* imaging of tumor macrophages revealed that in contrast to tumor periphery these cells are highly mobile within the tumor microenvironment, exhibit structural diversity and gene expression profile that includes increased ARG1. The number of these ARG^+^ macrophages significantly decreased after anti-PD-1 monoclonal antibody treatment (115). TAMs in lung cancer and melanoma also express more ARG1 than all other cells within tumor combined (178) and have over 20 times higher expression of ARG1 as compared with peritoneal macrophages (184).

Arginase production by macrophages not only leads to the inhibition of anti-tumor response *via* L-arg degradation, but also increases the proliferation of tumor cells, which is associated with the production of L-ornithine and then a polyamine—putrescine that promote tumor cells proliferation (185). Moreover, L-arg depletion in the tumor microenvironment attenuates NO production and reduces its cytotoxic effects on tumor cells (185). Several studies also indicate that arginase activity might be associated with delivery of additional metabolites with immunosuppressive properties. For example, inhibition of polyamines synthesis together with blocking of dietary polyamine transport was shown to exert antitumor effects that were associated with decreased numbers of intratumoral MDSCs and increased numbers of T-cells (186). Similar approach was shown to increase in granzyme B^+^IFN-γ^+^CD8^+^ T-cells and a decrease in immunosuppressive tumor-infiltrating cells including PMN-MDSCs, Tregs, and M2 macrophages (187).

### Neutrophils

Neutrophils are the most abundant leukocytes in peripheral blood and are produced in the bone marrow at a prodigious rate of 1 × 10^11^ cells per day (188). These cells constitute a rapidly reacting part of innate immune response, playing important role in defense against bacteria and fungi. Despite their important role in host defense, the increased numbers of neutrophils in blood of cancer patients correlate with poor prognosis (189). These cells can also be found in tumors, but their role in tumor has been largely neglected, mainly due to the belief that their life-span is one of the shortest among all leukocytes. However, tumor-associated neutrophils (TANs) persist in tumor microenvironment for extended time in response to GM-CSF and TGF-β (126). TANs are divided into two subtypes: N1 and N2, with anti-tumor and protumorigenic phenotype, respectively, but to date no specific molecular surface markers have been identified to distinguish them. Nonetheless, N2 neutrophils are characterized by high arginase expression (132, 190). ARG1 is in fact constitutively expressed in human neutrophils. However, these cells do not metabolize L-arg (123) possibly due to the confinement of ARG1 in gelatinase granules (191). Neutrophils can release ARG1 leading to the suppression of T-cells function (192). This process requires simultaneous exocytosis of ARG1-containg gelatinase granules and azurophil granules (192). It was assessed that 1 × 10^6^ of neutrophils secrete ARG1 at amounts sufficient to catabolize all the L-arg contained in 5 ml of blood in 1 h (193). At least in some tumors ARG1^+^ neutrophils are quite abundant and the presence of ARG1^+^ neutrophils correlates with suppressed T-cell functions (193, 194). Intriguingly, in non-small cell lung cancers despite high arginase activity in tumor microenvironment, most of the TANs display low or no ARG1 expression, in contrast to neutrophils in peritumoral tissue that strongly stain for ARG1 (193). It turned out that tumor cells release IL-8 that induces ARG1 exocytosis from neutrophils into extracellular milieu (193) (Figure 4). Degranulated neutrophils are also expanded in peripheral circulation of cancer patients, and ARG1 released from these cells strongly contributes to general suppression of T-cell functions (195). ARG1 released from neutrophils has also been shown to inhibit the proliferation of NK cells and IL-12/IL-18-induced production of IFN-γ (196). Zoledronic acid, a bisphosphonate used in the treatment of osteoporosis has been shown to induce ARG1 in neutrophils that suppress the activity of γδ T-cells (197). All these observations indicate that ARG1^+^ neutrophils seem to play a detrimental role in tumor progression, mainly due to immunosuppressive effects. Notably however, a recent study indicated that high intratumoral neutrophil numbers expressing ARG1 correlate with better survival of patients with colorectal cancer (198).

### Dendritic Cells

Dendritic cells (DCs) are classically described as professional antigen-presenting cells that produce cytokines and provide co-stimulatory molecules, leading to naïve T-cells activation and differentiation into effector cells (199). There are conventional DCs (cDCs), plasmacytoid DCs (pDCs), and monocyte-derived DCs (MoDCs) that have different origin and differ in function. Within cDCs there are additional subsets both in mice and in humans that are referred to as cDC1 and cDC2. cDCs1 are presumed to be primarily involved in cross-presentation of antigens to CD8^+^ T-cells, while cDCs2 seem to be largely associated with stimulating CD4^+^ T-cells (200). Another layer of subdivision into migratory and lymph node (LN)-resident DCs reflects location and the mechanisms of antigen acquisition by these cells. Migratory CD103^+^ DCs take up antigens in non-lymphoid tissues (including tumors) and traffic through lymphatic vessels into LNs. LN-resident CD8αα^+^ DCs enter the LNs from the blood and acquire antigens draining through the lymphatics or transported to LNs by other cells (200).

Tumors are frequently infiltrated by various populations of DCs. During infections DCs acquire, process and present antigen in association with MHC molecules, deliver co-stimulatory signals and release cytokines that shape T-cell responses. The same role is expected to be played by DCs in tumors. However, the stimulatory activity of these cells is often compromised and tumor DCs often drive tolerance rather than immunity in cancer patients (201). The mechanisms of tumor-infiltrating DCs that hamper development of antitumor immune response include decrease in MHC class I and II levels as well as in co-stimulatory molecules (CD40, CD80, CD86), rise in co-inhibitory molecules (such as PD-L1, PD-L2, VISTA), increased tryptophan degradation by indoleamine 2,3-dioxygenase (IDO1), decreased release of IL-12, but increased secretion of IL-10 and TGF-β, among others (201). Arginases can be added to this expanding list, based on numerous reports.

Lung cancer cells isolated from murine tumors induced DCs to differentiate into regulatory cells that suppressed T-cell response through ARG1 (202). In another study tumor-infiltrating DCs were observed to decrease the expression of CD3ζ in T-cells in ARG1-dependent manner and induced anergy in naïve CD8^+^ T-cells (203). ARG1 produced by DCs promotes the generation of FoxP3^+^ Tregs (204, 205). Not only ARG1 was shown to be expressed by DCs. Human fetal cDC2 cells uniquely express constitutively high levels of ARG2, through which these cells inhibit T-cell activation and TNF-α release (206).

The expression of ARG1 in DCs is regulated by a number of cytokines and tissue factors (Figure 4). As in other myeloid cells, ARG1 is induced by type 2 cytokines, including IL-4 and IL-13. Tregs were reported to induce ARG1 in DCs in a TGF-β-dependent mechanism (207). Supernatants from tumor cells experiencing endoplasmic reticulum (ER) stress and unfolded protein response (UPR) was shown to induce ARG1 in DCs (208). Retinoic acid was also shown to be a key mediator regulating expression of ARG1 in DCs, mediated by retinoic acid-responsive elements in the 5′ non-coding region of the ARG1 gene. Blockade of retinoic acid receptors makes DCs less responsive to IL-4 and GM-CSF (205).

## Mechanisms of Immunoregulatory Function of Arginase

An obvious question in understanding the role of amino acid-degrading enzymes in the regulation of the immune response is why do myeloid cells degrade L-arg Perhaps the best answers come from studies in mice with targeted deficiency of ARG1 in myeloid cells and the regulation of immune response and inflammation triggered by infectious microorganisms. ARG1 induced in macrophages during *Schistosoma mansoni* infection prevented cachexia, neutrophilia, and endotoxemia during acute schistosomiasis. Moreover, ARG1^+^ macrophages promoted TGF-β production and Foxp3 expression, suppressed antigen-specific T-cell proliferation, and limited Th17 differentiation. In mice with deficiency of ARG1 in myeloid cells infection with *Schistosoma mansoni* triggered a lethal T-cell-dependent immunopathology with non-resolving inflammation (209). On the other side, ARG deficiency in myeloid cells results in substantially decreased tumor growth (210) and increased CD8^+^ T-cells numbers and activity as compared with wild-type mice (211).

### Effects on Effector Functions in T-Cells

Lack of any single essential amino acids restricts T-cells activation and proliferation and this phenomenon is not specific to L-arg. Depletion of L-histidine, L-leucine, L-lysine, L-phenylalanine, L-threonine, and L-valine inhibited the proliferation of T-cells to a similar extent as L-arg depletion (207). Of importance, however, only arginases as well as IDO that hydrolyzes L-tryptophan (212, 213) are substantially increased in cancer.

#### Role of L-arg in T-Cell Proliferation

**Figure 5:**
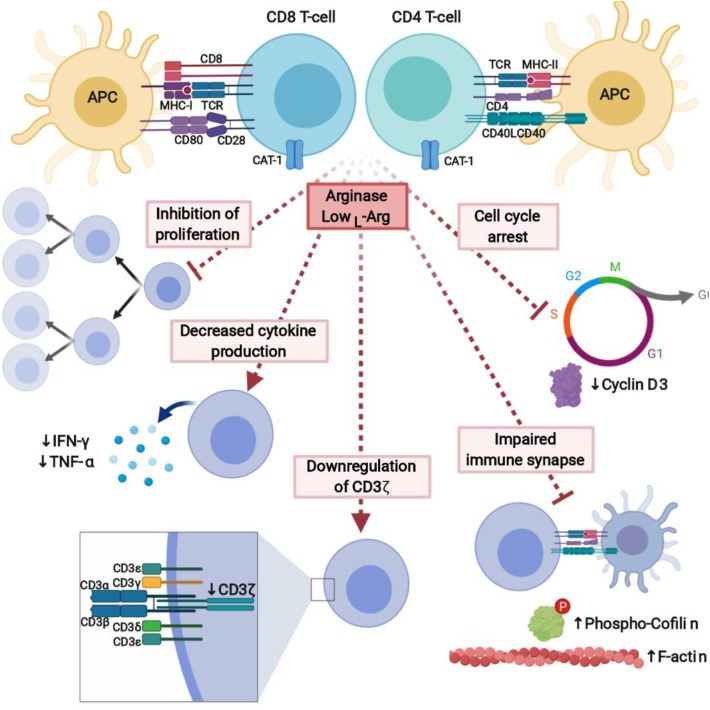
L-Arginine depletion by arginase potently inhibits immune response. Lack of L-arg completely inhibits proliferation of T-cells and leads to the decreased cytokine production. It is caused by downregulation of signal-transducing CD3ζ chain, cell cycle arrest, and affected formation of the immune synapse between T-cells and APC. Created with BioRender.

One of the hallmarks of ARG activity in the immune system is impaired T-cell proliferation (Figure 5). Proliferation of both human and murine T-cells is completely inhibited in L-arg-free medium after stimulation with anti-CD3- and anti-CD28-coupled beads or different types of mitogens. A similar inhibition of the T-cells proliferation is also triggered by ARG-producing cells, and this effect is restored by L-arg supplementation or arginase inhibitors (123, 132, 203, 214, 215). It is of note that T-cells remain viable in L-arg-depleted medium (123) and resume proliferation as soon as L-arg is added to the culture medium. The minimum L-arg concentration in cell culture medium necessary for one division of murine T-cell was determined to be 23 μM (216). Upon activation, when large amounts of L-arg are needed, T-cells rely mainly on the extracellular L-arg transport. A potent increase in the expression of cationic amino acid transporter-1 (CAT-1) is observed in both naïve and memory CD4^+^ and CD8^+^ T-cells after activation (22). Silencing of CAT-1 expression leads to the inhibition of T-cell proliferation, but not impaired TNF-α, IFN-γ, IL-2, IL-6 production (22).

#### Role of L-arg in T-Cell Cytokine Production

Secretion of several cytokines that play a critical role in T-cell differentiation and effector functions is also diminished in L-arg-starved cells (Figure 5). Conspicuously, this especially refers to the secretion of Th1 cytokines, including IFN-γ and tumor necrosis factor β (TNF-β) (123, 214, 217), although T-cells cultured in L-arg-free medium also secrete lower amounts of IL-5, and IL-10 as compared with T-cells cultured in complete medium (218). The decrease in IFN-γ secretion is also induced by ARG^+^ tumor-infiltrating DCs (203) and ARG inhibitors administered *in vivo* increase IFN-γ secretion (219). On the contrary, the synthesis of IL-2, IL-6, and IL-8 seems to be unaffected by the absence of L-arg (217), although in another study PMN-MDSCs were shown to suppress IL-2 production from T-cells and this effect was restored by ARG inhibitor (220).

#### Role of L-arg in T-Cell Differentiation

Upon antigen recognition naïve T-cells proliferate and acquire effector functions that are dependent on multiple additional signals delivered in the microenvironment of secondary lymphoid organs. The signals include various cytokines, growth factors, and surface-associated molecules (including co-stimulatory and adhesion molecules) (221, 222). Accumulating evidence indicates that L-arg metabolism plays an important role in regulating T-cell differentiation. For example, oral administration of L-arg in a mouse model of breast cancer increased the levels of T-bet, a transcription factor associated with Th1 cells (223). Moreover, it increased the frequency of CD8^+^ T-cells, and mRNA levels of granzyme B and IFN-γ in the tumor (223). High extracellular L-arg increased the survival of T-cells stimulated with IL-2 after cytokine withdrawal and favored the formation of central memory T-cells (224). Inhibition of L-arg transport into the cell decreased T-cell longevity further confirming the role of L-arg in human T-cells survival (224).

Somewhat enigmatic and to some extent contradictory reports refer to L-arg metabolism and Treg cells development. In an interesting study FoxP3^+^ Tregs were shown to induce ARG1 (as well as other enzymes involved in amino acid metabolism) in DCs, thereby increasing amino acid consumption in local microenvironment. This reduced mTOR signaling and favored development of additional Tregs (207). Inhibition of mTOR signaling by rapamycin or amino acid depletion was shown to induce FoxP3, but L-arg deficiency was effective only when TGF-β was added (207). Moreover, ARG2 was found in Tregs from normal skin and its expression increased in metastatic melanoma (225). ARG2 in Tregs was demonstrated to attenuate mTOR activity and conferred Tregs with enhanced suppressive activity (225) suggesting that low intracellular L-arg concentrations may facilitate Tregs development. Consistently, with these findings, T-cells from mTOR-deficient mice preferentially become regulatory, but not effector T-cells (226).

However, another study showed that mice fed with L-arg-deficient diet had modestly reduced number of peripheral effector Tregs and these cells had reduced expression of ICOS and CTLA4. L-arg turned out to be essential for sustaining mTORC1 activity, functional programming, and Treg cell-mediated immunosuppression (227). Moreover, disruption of mTORC1 in FoxP3^+^ T-cells caused a loss of Treg suppressive activity *in vivo* and led to development of systemic immunopathology in mice indicating that Treg cell responses are critically dependent on mTORC1 signaling (228). Clearly, the effects of L-arg metabolism on T-cell differentiation are very complex and require further studies, especially that still another report indicated that ARG1 in MDSCs is participating in promotion of Th17 differentiation (229).

### Molecular Mechanisms of Immunoregulatory Effects Associated With L-arg Metabolism

The exact molecular mechanisms of L-arg starvation-mediated immunosuppression still remain to a large extent enigmatic. Up to now, L-arg starvation was shown to affect T-cell antigen receptor ζ chain (CD3ζ) expression (230) and phosphorylation of other signal-transducing proteins (231), and therefore to impair transduction of activation signal, cell cycle progression (232), as well as formation of the immune synapse (231) (Figure 5).

#### Downregulation of the CD3ζ and Impaired Signal Transduction

The main mechanism by which L-arg starvation inhibits T-cells proliferation is through downregulation of the CD3ζ chain (230, 233). CD3ζ is a critical component of the TCR complex that couples antigen recognition to the intracellular signaling pathways (234). After T-cells stimulation, TCR proteins including CD3ζ undergo internalization followed by re-expression, externalization, or sorting to lysosomes for degradation (235, 236). A common finding in cancer patients is a marked decrease in the expression of CD3ζ in T-cells (143, 237).

Many studies reported that L-arg depletion in culture medium leads to a rapid decrease of CD3ζ levels (132, 230, 238). Of note, the changes in TCR receptor subunits expression during L-arg starvation are observed only in stimulated T-cells (218). This decrease is specific to L-arg-starvation, since lack of glutamine or leucine (233) as well as glycine or lysine (218) did not change the levels of CD3ζ. However, a decrease in CD3ζ was also reported to be caused by hydrogen peroxide secreted from tumor macrophages (239). The decrease in CD3ζ is completely reversed by L-arg supplementation in cell medium (230) or ARG inhibition when co-culture with ARG-producing cells is used (132). A similar downregulation of CD3ζ and CD3ε levels is induced by tumor-associated myeloid cells, which express ARG1 (132). This effect is prevented by the addition of ARG inhibitor (N-hydroxy-nor-L-arg) or L-arg supplementation, but not by the catalase, a hydrogen peroxide scavenger (132), as suggested before (239).

How L-arg starvation selectively impairs CD3ζ expression still remains unclear. L-arg starvation of human T-cells did not affect the degradation of CD3ζ in proteasome or lysosomes (218). Therefore, it was suggested that L-arg depletion may impair CD3ζ synthesis (218) or the stability of mRNA for CD3ζ (230).

#### Cell Cycle Arrest

Another defined mechanisms by which L-arg starvation restricts T-cells activation and proliferation is the regulation of cell cycle progression (232) *via* modulation of cyclin D3 mRNA stability (240). Cyclins, including cyclin D3 (241), are critical regulators of the cell cycle, immune cells development and proliferation (242). L-arg starvation arrests human T-cells in G_0_-G_1_ phase (232). The levels of cyclin D3 as well as CDK4 significantly increase after T-cells activation, however, not in absence of L-arg. Moreover, silencing of cyclin D3 in Jurkat cells reproduces effects induced by L-arg starvation (232). Cyclin D3 was shown to be regulated by L-arg through transcriptional, posttranscriptional, and translational mechanisms (232). In the absence of L-arg human T-cells have decreased phosphorylation of the retinoblastoma protein (Rb), which is the major substrate for the cyclin D/cyclin-dependent kinase complex, as well as decreased levels of E2F-1, which is crucial for the initiation of the transcription of genes involved in the G_2_/S transition (232). In the absence of L-arg there is a global arrest in *de novo* protein synthesis. L-arg starvation also affects the expression of HuR, RNA binding protein, that stabilizes mRNA of cyclin D3 by the binding to the 3'-untranslated region (UTR) and shuttles its transport to the cytoplasm. Silencing of HuR exerts similar effect on T-cells proliferation as L-arg starvation (240).

#### Changes in the Immune Synapse Between APC and T-Cells

Proliferation of T-cells after antigen presenting cells (APC)-based cellular activation is also completely inhibited in the absence of L-arg (231). The formation of immune synapse between T-cell and APC is critical for the activation of effector cell (243). In L-arg-depleted medium, the formation of the immune synapse is impaired. T-cells activated in the absence of L-arg have increased F-actin concentration, which may be caused by impaired cofilin dephosphorylation (231). Cofilin is a small actin-remodeling protein that couples T-cell activation via the TCR and co-stimulatory receptors in the immune synapse (231, 244). Phosphorylation of ERK1/2 is significantly reduced in L-arg absence, however, the phosphorylation of AKT is increased to the higher level compared to the cells activated in L-arg-containing medium (231). It leads to the impaired dephosphorylation of cofilin that results in impair immune synapse formation. Impaired dephosphorylation of cofilin in human T-cells was also induced by cell-free human pus supernatant, which is known to contain high arginase activity (123). This effect may be prevented by arginase inhibitor (231).

#### L-arg in Metabolic Regulation of T-Cells

Proliferation and differentiation of T-cells can occur only if sufficient access to metabolites and nutrients is ensured (245). A recent metabolomic analysis of activated T-cells revealed that out of 429 measured metabolites only 14 were less abundant in activated T-cells, and L-arg was the only protein amino acid among them (224). A drop in intracellular L-arg levels was observed despite induction of CAT-1 transporters. Interestingly, the intracellular levels of L-glutamine, which is also intensively metabolized in activated cells, remained high. Along with CAT-1 induction, T-cell activation was associated with increased expression of L-arg metabolism-related enzymes including ARG2, OAT, and spermidine synthase (SRM). Once entering the cell, L-arg turned out to be rapidly converted into L-ornithine, agmatine, and putrescine. Importantly, increasing L-arg concentration in the culture medium upregulated gluconeogenesis-related genes, serine biosynthesis pathway, and mitochondrial tricarboxylic acid cycle, while downregulating glucose transporter and glycolytic enzymes. These changes promoted mitochondrial OXPHOS in activated T-cells, while downregulating glycolysis (224). Global analysis of T-cells proteome changes in response to high L-arg concentration revealed several proteins that are responsible for increased T-cells survival. These can be assigned into four functional groups, including mRNA splicing, DNA repair mechanisms, regulation of the cytoskeleton and the ribosome (224).

Oral supplementation of L-arg that increased its serum concentration over 4-fold allowed more robust induction of antigen-specific T-cell proliferation in mice. Moreover, T-cells from ARG2^−/−^ mice, incubated with supplemental L-arg or treated with ARG inhibitor reveled much better survival after cytokine withdrawal (224). In a complementary study, CD8^+^ T-cells from ARG2-deficient mice showed markedly superior antitumor activity in mice and turned out to respond stronger to PD-1 blockade as compared with ARG2^+^ T-cells (246). Moreover, ARG2-deficient T-cells were characterized by faster acquisition of effector functions, increased persistence and enhanced differentiation into memory cells.

Altogether, these studies indicate that ARG2 might be a metabolic gatekeeper in T-cells. In activated T-cells ARG2 degrades L-arg and generates agmatine and polyamines. In case of accessible L-arg in the extracellular environment the intracellular pool of this amino acid can be replenished. However, at sites, where extracellular L-arg is depleted (by tumor cells or tumor-infiltrating myeloid cells) the intracellular pool cannot be restored leading to T-cell suppression (224).

Mechanisms of arginine-starvation sensing in immune cells are still unclear. It is suggested that mTOR together with GCN2 kinase regulate amino acid metabolism and response to arginine starvation (207, 227, 232, 247, 248), however, the exact mechanism is unknown and requires further investigation.

### B Cells and L-arg

The role of L-arg in B-cells functions was much less investigated and is poorly understood. It was shown that L-arg deficiency due to high ARG1 activity in F/A-2^+/+^ transgenic mice, that overexpress arginase in enterocytes, potently impairs early B cell maturation with no major impact on T-cells (249). F/A-2^+/+^ mice have reduced number of B cells, decreased serum IgM concentration and hampered B cell maturation in the early pre-B cell stage (249). L-Arg-free diet fed mice which have significantly lower concentration of plasma L-arg compared to L-arg-supplemented diet had also impaired antigen-specific mucosal immune response against tetanus toxoid (TT). After oral administration, no TT-specific fecal IgA antibodies were detected in L-arg-free diet fed mice (250). Both PMN-MDSCs and M-MDSCs were shown to regulate key B-cell functions, particularly B-cell proliferation and antibody production. PMN-MDSC-mediated B-cell suppression turned out to be cell contact dependent and involved ARG1 (251). A recent study from the same group indicated that M-MDSCs suppress B-cell proliferation, and downregulate IgM, HLA-DR, CD80, CD86, TACI, and CD95 in contact independent, but ARG1 and iNOS-dependent mechanism (252).

### Myeloid Cells and L-arg

The role of L-arg in differentiation of myeloid cells is poorly investigated. Most of the studies focused on the role of ARG1 produced by myeloid cells rather than the dependence of these cells on L-arg. Individual results *in vitro* show no influence of L-arg on macrophages differentiation, maturation, and effector functions. In the absence of L-arg, maturation of macrophages into classically activated macrophages (M1) and alternatively activated macrophages (M2) was unaffected (253). Moreover, the production of cytokines by both macrophage subtypes was unimpaired under L-arg-starvation (253). Likewise, the expression of iNOS by M1 cells as well as the expression of ARG by M2 cells turned out to be independent from the L-arg concentration (253). However, ARG1 expression was essential for monocytic DC differentiation (254). ARG1 was also recently shown to be crucial in efferocytic clearance of apoptotic cells by macrophages (255).

*In vivo* however, L-arg supplementation was shown to promote Gr-1^+^CD11b^−^F4/80^+^, but suppressed Gr-1^+^CD11b^+^F4/80^+^ macrophages in a murine model of breast cancer (223). However, these effects might not be caused directly, but rather result from the effects on T-cells activation. Another study showed that L-arg starvation promotes tumor G-MDSC accumulation, which further suppress T-cells anti-tumor response (158). Similar results were obtained with PEG-asparaginase administration, suggesting that generally amino acid starvation results in MDSC accumulation. PEG-ARG1-induced MDSC accumulation was found to be regulated by GCN2, since the accumulation of MDSCs in GCN2-deficient mice treated with PEG-ARG1 was negligible (158). Importantly, MDSCs isolated from GCN2-deficient mice had similar immunosuppressive properties as compared with MDSCs isolated from wild-type mice, which suggests that GCN2 is involved in the accumulation of MDSC, but not in their effector functions. Moreover, it was observed that ARG2-releasing AML blasts as well as ARG2-rich plasma of patients with AML promotes the differentiation of monocytes toward M2 macrophages. These effects were diminished by L-arg supplementation or arginase inhibitors (61).

### NK Cells and L-arg

NK cells are less sensitive to low L-arg concentrations as compared with T-cells however, L-arg starvation affects the main effector functions of NK cells (196). L-arg starvation decreases NK cells proliferation and viability, as well as cytotoxic activity (210, 256). Depletion of L-arg leads to the reduction in the expression of NKp46 and NKp30 activating receptors, as well as the NK cell ζ chain expression in the FcγRIIIA, similar to the CD3ζ chain in T-cells. Moreover, in the absence of L-arg the production of IFN-γ by NK cells is significantly decreased (256). Similar effect is exert by arginase from human neutrophils (196). However, NK cell degranulation and cytotoxicity seems to be unaffected by L-arg depletion (196).

### L-arg Metabolites and Immune Response

L-arg is in the center of many metabolic pathways. Arginase not only depletes L-arg, but also creates multiple downstream metabolites including L-ornithine and urea, as well as L-proline, glutamate, agmatine, putrescine, L-citrulline, and polyamines.

#### Ornithine

L-arg is degraded by arginase to L-ornithine and urea. While the concentration of L-arg substantially decreases in cancer, the concentration of L-ornithine increases (59, 75, 257). High concentration of L-ornithine in tumor interstitial fluid may inhibit anti-tumor cytotoxic response of CD8^+^ T-cells (258, 259), and together with L-arg depletion, that affects T-cells properties but not cytotoxicity (214), provide effective tumor evasion of the immune system. Reversible inhibition of cytotoxicity of T-cells in the presence of L-ornithine is independent from the type of stimulation and it seems that it affects early stages of CTL activation (258). However, L-ornithine did not impair mitogenic response to the stimulation (258, 259), as well as IL-2 and IL-3 production (258). ODC catalyzes the conversion of L-ornithine to polyamines.

#### Polyamines

A diamine putrescine, triamine spermidine, and tetraamine spermine are ubiquitous L-ornithine metabolites associated with important cellular processes. Polyamines are essential for cell growth and proliferation during development, wound healing, and tissue regeneration. ODC catalyzes the conversion of L-ornithine into putrescine, which is then metabolized to spermidine by spermidine synthase and spermine by spermine synthase (260). At physiological pH polyamines are positively charged and bind to acidic sites in DNA and RNA, controlling gene expression (261). Moreover, polyamines have antioxidative properties, bind to K^+^ channels, NMDA receptors, and modulate the activity of various enzymes (261).

Growth promoting functions of polyamines are best described in tumors. However, it seems that polyamines are also important in T-cell clonal expansion. It has been suggested that the synthesis of polyamines in T-cells is under the direction of Myc, as Myc-deficient T-cells fail to induce ODC and other genes involved in polyamine synthesis, leading to decreased polyamine production (262). Spermidine is also a precursor of hypusine, which post-translationally binds to eukaryotic initiation factor 5a (eIF5a). Intriguingly, eIF5a, which prevents ribosomal stalling during translation of certain mRNAs, is one of the most strongly expressed proteins in activated T-cells (263).

Polyamines were reported to exert anti-inflammatory effects in macrophages by restraining activation of M1 while promoting differentiation of M2 subtype. For example, LPS-induced expression of TNF, IL-1, IL-6, IL-12, iNOS, and CD80 was suppressed by polyamines (264–266). Polyamines also modulate immunoregulatory activities of DCs. IDO1 activity in TGF-β-treated DCs requires ARG-1-dependent spermidine synthesis that activates Src tyrosine kinase, which participates in IDO1 phosphorylation (267).

## Arginase Inhibitors

Expanding knowledge on the biological role of arginases prompts the idea of therapeutic inhibition of these enzymes. The interplay between ARG and NOS resulting mainly from the competition for the common substrate L-arg makes ARG inhibition an attractive approach in the treatment of cardiovascular and inflammatory conditions (such as asthma, diabetes, hypertension, atherosclerosis, coronary artery disease, heart failure or erectile dysfunctions). Furthermore, inhibition of immunosuppressive functions of arginases is being explored in the treatment of cancer. Modulation of L-arg metabolism is also being explored as a therapeutic strategy in Alzheimer's disease (268).

As many pathogenic bacteria (such as *Helicobacter, Mycobacterium, Salmonella*), fungi (*Candida*) and parasites (*Trypanosoma, Leishmania, Schistosoma*) express species-specific isoforms of ARG to facilitate their survival in the host, finding pathogen-ARG-specific inhibitors emerges as a timely approach in the antibiotic-resistance era. Interestingly, *Leishmania* parasites induce ARG1 expression in infected macrophages to decrease L-arg availability for iNOS and thus to avoid NO toxicity (269). The latter observation further supports the potential use of ARG inhibitors in the treatment of infectious diseases.

Currently, almost all ARG inhibitors being developed as drug candidates are competitive inhibitors of both isoenzymes (ARG1 and ARG2) and in vast majority are L-arg analogs (270). Finding an isoform-specific ARG inhibitor is challenging as 100% homology exists in the active site between human ARG1 and ARG2. As the results of the preclinical, mainly *in vitro*, testing of ARG inhibitors have been extensively reviewed elsewhere (270, 271), here we just briefly summarize the data on *in vivo* and clinical activity of selected ARG inhibitors.

So called first generation of ARG inhibitors such as N-hydroxy-nor-L-arginine (nor-NOHA) (272), (S)-2-amino-6-boronohexanoic acid (ABH) (273) and (S)-(2-boronoethyl)-L-cysteine (BEC) (274) are reversible, modest inhibitors of ARG1 and ARG2 enzymatic activity with either poor pharmacokinetic properties or insufficient penetration through the plasma membrane. In mouse models nor-NOHA has been shown to inhibit local tumor growth in B- and T-cells-dependent manner as well as to reduce metastatic burden (132, 178, 275). Second generation compounds are characterized by better pharmacokinetic and pharmacodynamic properties. As an example, so called compound 9 [(R)-2-amino-6-borono-2-(2-(piperidin-1-yl)ethyl)hexanoic acid] has been recently showed to decrease the growth of KRAS mutated murine lung tumors via inhibition of ARG activity in tumor-infiltrating myeloid cells (117).

Up to date, there are only two ARG inhibitors being tested in clinical trials. Both drug candidates have been developed by Calithera Biosciences and are orally available small-molecule compounds. INCB001158 (CB-1158) is being evaluated in Phase 2 as a single agent and in combination with immune checkpoint inhibitors in cancer (both solid tumors and multiple myeloma), while CB-280 in Phase 1 in cystic fibrosis, exploiting the novel idea of increasing NO production to improve lung function. CB-1158 has been shown *ex vivo* to reverse human T-cell immunosuppression mediated by ARG1 produced by neutrophils as well as MDSCs (210). It also exerts immune-based antitumor effects in syngeneic mouse tumor models *in vivo* as a single agent as well as in combination with the immune checkpoint inhibitors (210). An interesting ARG inhibitor to watch is OATD-02 (276), a compound being developed by Oncoarendi Therapeutics. In preclinical models it has been shown to delay ovarian cancer progression and to revert ARG1-mediated inhibition of antigen-specific T-cells proliferation and to restore their CD3ζ levels (64). Moreover, in syngeneic mouse tumors it potentiated the antitumor efficacy of immune checkpoint inhibitors (277). The company claims OATD-02 Phase 1 trial in cancer patients to begin in 2020-2021.

Arginase inhibition cannot be replaced, however, by chronic L-arg supplementation. Dietary intake of L-arg results only in a transient increase of L-arg plasma concentration (278). Moreover, if arginases are active in blood or body tissues, it is very likely that they easily degrade the excessive amounts of this amino acid.

Global ARG1 inhibition rises significant safety concerns. ARG1 gene knockout mice die 10–14 days post-birth (39). Similarly, induction of whole body *Arg1* KO in adult “floxed” *Arg1 CreET*^*T*2^ transgenic mice leads to the animals death in up to 2 weeks post-tamoxifen administration (279). The major cause of death in *Arg1* KO mice is hyperammonemia resulting from the defect of the liver urea cycle. It is the lack of *Arg1* expression in the liver that is fatal, as hepatocyte-specific knockout of *Arg1* mimics the whole body deficiency of this enzyme (280). Lack of *Arg1* expression leads to altered hepatocytes morphology, significantly increased plasma L-arg and L-citrulline concentrations accompanied by decreased plasma L-ornithine and L-proline concentrations (39). Interestingly, *Arg2* knockout mice are viable and do not have a disabling phenotype apart from high plasma L-arg concentrations and decreased male fertility. Moreover, *Arg2* KO mice have significantly extended lifespan, indicating some role of this enzyme in aging (41). Double *Arg1* and *Arg2* KO mice show the same phenotype as *Arg1*-lacking animals. Unexpectedly, in *Arg1* KO mouse embryo no compensatory *Arg2* expression was observed (281), suggesting non-overlapping role of both arginase isoenzymes in murine embryonal development. In humans, ARG1 deficiency is a rare autosomal recessive disorder, resulting from over 40 reported mutations in *ARG1*. In the most severe form ARG1 deficiency results in hyperargininemia, neurological impairment and eventually fatal episodes of hyperammonemia (282). ARG1 deficiency is frequently accompanied by a compensatory increase in ARG2 activity in the kidney, ameliorating metabolic disturbances (44). The latter observation encourages a still very challenging attempt to develop ARG1-specific inhibitors.

Animal studies confirmed that there is a safe therapeutic window for tested ARG inhibitors. In both mice and rats, over 2-months long daily systemic administration of nor-NOHA did not result in detectable toxicity. It is likely, that due to the quantitative differences in ARG1 expression between the liver and other tissues way lower ARG inhibitors concentrations are needed to exert immunomodulatory and/or vascular effects than to block the Krebs cycle in hepatocytes (270).

Initial results of the investigational trial of the oral ARG inhibitor INCB001158 in colon cancer patients proved acceptable safety profile of this drug candidate. A maximum tolerated dose was not reached even for the twice daily total dose of 150 mg. Moreover, clinically significant urea cycle inhibition was not observed. In microsatellite stable (MSS) colorectal cancer patients involved in this study, 7 and 6% of partial responses to the INCB001158 and pembrolizumab (anti-PD1 monoclonal antibody) combination or INCB001158 monotherapy, respectively, were reported. Importantly, objective pharmacodynamic parameters such as an increase in the intratumoral CD8^+^ T-cells as well as dose-related increase in plasma L-arg were achieved in the treated individuals (283).

To evaluate the clinical efficacy of ARG inhibition in a comprehensive way we need much more data. Nonetheless, existing preclinical and initial clinical evidence seems to support the idea that therapeutic targeting of the immunomodulatory ARG might serve as a potent addition to the other immunotherapeutic strategies rather than as an effective single agent treatment. Moreover, it would be crucial not only to evaluate proper dosing, timing and treatment duration but also to find reliable biomarkers predicting desirable clinical effects.

Although recent data support the idea that ARG overexpression correlates with poor prognosis, a number of studies indicate that arginine depletion may also be beneficial for subgroups of patients, especially those with inactivation of ASS1 in cancer cells that leads to the dependence on exogenous L-arg (284). L-Arg deprivation by ADI conjugated with polyethylene glycol (ADI-PEG) (84, 285) as well as pegylated recombinant human ARG (rhARG-PEG) (286, 287) were applied to the treatment of arginine-auxotrophic tumors and showed potent anticancer effects [reviewed in (288)].

Noteworthy, L-arg-restriction as the regulation of immune response is not specific to the cancer. It was shown that *Helicobacter pylori* by arginase not only produces urea which can be used to CO_2_ and NH_3_ production by urease to support acid tolerance (289). *H. pylori* using ARG also depletes L-arg which leads to the downregulation of CD3ζ and inhibition of T-cells proliferation during infection (290). T-cells response is also suppressed *via* ARG by human embryonic stem cells (291). ARG also mediates T-cells hyporesponsiveness in human pregnancy (292), post-stroke immunosuppression (293), as well as in the control of autoimmunity (294). Moreover, *H. pylori* induces ARG2 expression in macrophages contributing to the immune evasion by limiting production of antimicrobial NO (48). Crucial role of ARG in the regulation of immune response by impairing NO production was also described in the model of cutaneous contact hypersensitivity (295) as well as in immune response to *Leishmania major* infections (296). Importantly, some intracellular pathogens induce expression of ARG1 in macrophages that hampers effective immune response (137). A recent study revealed that increased ARG levels may play a role in fatigue intensification in cancer patients undergoing external-beam radiation therapy (297)

## Final Remarks

ARG expression is substantially elevated in myeloid cells in cancer and mitigate antitumor response via multiple mechanisms. Intriguingly, cytotoxic effects of T-cells are unaffected by a lack of L-arg, despite the fact that CD3ζ and CD3ε are downregulated and thus TCR signal transduction should be inhibited. In contrast, T-cell proliferation is strongly suppressed, but it must be emphasized that T-cells proliferate extensively in tumor-draining lymph nodes, and not in the tumor. L-arg concentrations in tumor-draining LN have not been measured so far. It would also be interesting to see whether increased ARG activity contributes to fibrotic processes leading to desmoplastic changes in some types of tumors, such as pancreatic cancer. Increased activity of arginases could limit L-arg availability to NOS—could it be responsible for vascular abnormalities frequently described in tumorsxx Altogether, increasing evidence indicates that arginases become potentially important targets for therapeutic interventions that might improve the efficacy of immunotherapy, decrease infectious complications and improve quality of life of cancer patients.

## Author Contributions

All authors listed have made a substantial, direct and intellectual contribution to the work, and approved it for publication.

## Conflict of Interest

The authors declare that the research was conducted in the absence of any commercial or financial relationships that could be construed as a potential conflict of interest.
